# Urology never events in the United Kingdom: A retrospective 10‐year review

**DOI:** 10.1002/bco2.331

**Published:** 2024-02-01

**Authors:** Jerocin Vishani Loyala, Andrew Ang, Billy Down, Sarah A. Howles

**Affiliations:** ^1^ Oxford University Hospitals NHS Foundation Trust Oxford UK; ^2^ Nuffield Department of Surgical Sciences University of Oxford Oxford UK

**Keywords:** clinical governance, human factors, never events, patient safety

## Abstract

**Objectives:**

The aim was to assess the prevalence of never events (NEs) specific to urology in the United Kingdom and identify commonly occurring themes.

**Methods:**

Data from the National Health Service (NHS) NEs website were obtained and all NEs from 2012 to 2022 were reviewed. Urology‐specific NEs were identified and further analysed in their respective categories. Data regarding the total number of surgical procedures performed in the NHS specific to each specialty were obtained via the NHS Hospital Episode Statistics website.

**Results:**

There were 3972 NEs recorded over the 10‐year period with 95 (2.4%) of these as a result of urology surgery. The most common surgical intervention associated with a urological NE was ureteric stenting, which comprised 45/95 (47.4%) of all analysed NEs. These consisted of wrong site ureteric stent insertion (*n* = 29), wrong site ureteric stent removal (*n* = 9), wrong stent type (*n* = 5) and retained guidewires (*n* = 2). There were 7.14 million urology surgeries performed in the 10‐year period, and prevalence was 0.0013%.

**Conclusion:**

NEs are fully preventable serious incidents in the NHS. This is the first study to investigate the prevalence of NEs in urology in the United Kingdom. This study demonstrates that in the last 10 years the prevalence of urology NEs is low at 0.0013%, with ureteric stent procedures accounting for more than half of the NEs. Urologists should be mindful of the potential for wrong site surgery in urologic stenting procedures.

## 1 INTRODUCTION

Never events (NEs) are defined by National Health Service (NHS) England as fully preventable serious incidents that have available guidance or safety recommendations that should have been implemented by all healthcare providers.^1^ NEs have the potential to cause serious patient harm or death, but this is not a requirement for an incident to be categorised as an NE. In the United Kingdom, approximately 100 000 incidents per year are reported involving patient safety, of which 400–500 of these are considered NEs.^2^, ^3^

The most common NEs are wrong site surgery and retained swabs.^4^, ^5^ In an effort to minimise the occurrence of NEs, the World Health Organisation (WHO) implemented the widely used ‘Surgical Safety Checklist’, which has standardised communication among theatre staff globally. Improving preoperative planning and using surgical check list can reduce wrong site surgery.^6^, ^7^ Despite this, surgical specialities contribute significantly to the overall burden of NEs.^4^

To date, no publications have addressed the burden of NEs in urology in the United Kingdom or internationally. This is despite the potential of NEs to cause major patient harm; indeed, NEs result in death in up to 20% of cases.^8^ Furthermore, £74.5million was paid by the NHS over a 20‐year period as a result of litigation claims relating to urology.^9^

The aim of this study was to assess the prevalence of NEs specific to urology in the United Kingdom and to identify relevant themes that may help to mitigate their occurrence in future.

## 2 METHODS

It is mandatory to report all NEs within the UK NHS via the National Reporting and Learning System (NRLS) and Strategic Executive Information System (StEIS). NHS England collects the data and produces a yearly report for all NEs occurring in England. The NEs are classified according to various categories including wrong site surgery, retained foreign object post‐procedure and wrong implant/prosthesis. These are then subdivided into more specific categories.

All NEs on the NHS England database from 2012 to 2022 were analysed by a focus group of surgical trainees. This database covers NEs occurring in England, excluding Wales, Scotland and Northern Ireland. Urology‐specific NEs such as ‘wrong side ureteric stent’ or ‘cystoscopy intended for another patient’ were identified from the sub‐categories listed in the database. These urology‐specific NEs were reviewed and recommendations made under the supervision of a urology consultant.

There have been several changes made to the NEs reporting process. In 2015, the NE policy and framework were changed, and the definition of an NE changed to include all cases with potential to cause harm/death as opposed to actual harm. The reports for April 2018 to March 2019 and 1 April 2020 to 31 March 2022 were still provisional reports, which were yet to be finalised at the time of data collection. Furthermore, the way NEs are to be reported is also due to change. A new system, Learn from Patient Safety Events (LFPSE), was introduced in 2021, which is intended to replace the NRLS by September 2023. These factors prevented effective year‐on‐year comparison of NEs, and thus, this was not attempted.

Data regarding the total number of urology procedures performed in the same time period (2012–2022) in England were obtained via NHS digital's Hospital Episode Statistics.^10^ Urology procedures were identified using codes for urinary (M01–M86) and male genital organs (N01–N35).

## 3 RESULTS

**FIGURE 1:**
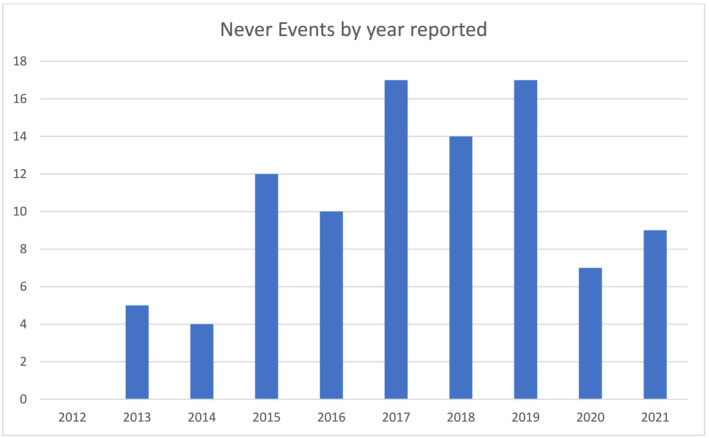
Urology‐specific never events by year reported.

A total of 3972 NEs were reported between 2012 and 2022. Of these, 3167 (79.7%) were procedural/surgical related. Urology‐specific NEs comprised 95 events (2.4%) and are detailed in Table 1. NEs displayed by year reported are included in Figure 1.

**TABLE 1:** Urology‐specific never events.

Wrong site/side	68
Ureteric stent insertion [wrong site]	29
Ureteric stent removal [wrong site]	9
Ureteroscopy +/− lithotripsy [wrong site]	13
Cystoscopy intended for another patient	8
Incision to wrong testicle [wrong site]	1
Excision of vas and testicular vessels [wrong site]	1
Epididymectomy [wrong site]	1
Exploration of wrong testicle [wrong site]	3
Urodynamic intended for another patient	1
Wrong aspect of kidney	1
Wrong nerve preserved during prostatectomy	1
Retained foreign body	**19**
Bladder resectoscope tip	4
Guidewire tip from urinary catheter	2
Guidewire from ureteric stent	2
Urological instrumentation [unspecified]	2
Guidewire from suprapubic catheter	1
Part of bladder instrumentation	1
Part of suprapubic catheter	1
Guidewire from urodynamics sensor	1
Sheath from ureteric balloon dilator	1
Guidewire from urethrotomy	1
Vasectomy clamps	1
Part of ureteric catheter	1
Urethral introducer fragment	1
Wrong implant/prosthesis	**5**
Wrong type of ureteric stent	5
Wrong procedure	**3**
Circumcision instead of frenuloplasty	1
Circumcision instead of flexible cystoscopy	1
Orchidectomy instead of epididymectomy	1

The most common surgical intervention associated with a urological NE was ureteric stenting, which comprised 45/95 (47.4%) of all analysed NEs. The most common NEs were both stent related: wrong site ureteric stent insertion (*n* = 29; 30.5%) and wrong site ureteric stent removal (*n* = 9; 9.5%). There were also two retained guidewires used in ureteric stenting and five wrong type of stents used.

NEs are categorised by the NHS into ‘Wrong site/side’, ‘Wrong procedure’, ‘Retained foreign body (FB)’, and ‘Wrong implant/prosthesis’. The most common of these types was wrong site surgery (65/95; 68.4%). Wrong site surgery was categorised into wrong site ureteric stent procedures (38/95), ureteroscopy +/− lithotripsy (13/95) and testicular surgery (6/95), cystoscopy meant for another patient (8/95) and other procedures (3/95).

Retained foreign body represented 20% of urological NEs (19/95), of which bladder resectoscope tip (4/19), guidewire from ureteric stent (2/19) and guidewire from urinary catheter (2/19) were the most common. The remaining FBs were single cases. There were five cases (5/95) of wrong implant/prosthesis, of which all were wrong type of ureteric stent, and three cases of wrong type of surgery (3.2%), all of which were testicular or penile surgeries.

There were 7.14 million urology‐specific procedures performed from 2012 to 2022 in England, based on NHS Digital data. The prevalence of NEs in England over the last 10 years is therefore 0.0013%.

## 4 DISCUSSION

We identified 95 NEs in the NHS relating to urology between 2012 and 2022, representing 0.0013% of all urological procedures. There has been a notable increase in NE reported comparing pre‐2015 and post‐2015 (Figure 1). This is likely due to changes made in 2015 in which a more robust process was introduced by NHS England in an effort to standardise and increase reports of NEs.^7^ A similar study of patients over a 9‐year period identified 797 NEs in general surgery, a higher number than our study. However, this study included generic NEs such as retained surgical swabs, wrong finger surgery and cervical biopsies; this may account for the differential NE rates.^5^ In contrast, a study of trauma and orthopaedic surgery NEs that restricted analyses to trauma and orthopaedic‐specific outcomes identified 460 NEs between 2012 and 2020, the most common of which were wrong implant/prosthesis (206/460) followed by wrong site surgery (197/460).^10^ Considering a total of 7.81 million trauma and orthopaedic procedures over this time (based on NHS Digital data), this represents 0.01% of all cases; an NE rate significantly higher than that of urology NEs (chi‐squared *p* < 0.001). These higher rates may be due to the increased risk of error when dealing with multiple limbs and digits as opposed to the renal system.

In our data, urology‐specific NEs comprised 2.4% of the total number of NEs reported during the study period. The most common type of urology NE identified was wrong site surgery, representing 65/95 (68.4%) of urological NEs. Precautionary principals have been made available including the WHO ‘Safe Surgery Checklist’ that aim to facilitate safe operative practices and prevent wrong site surgery and retained foreign bodies. However, a number of studies have demonstrated that NEs continue to occur.^4^, ^8^, ^9^ Practices such as utilising radiopaque markers to indicate surgical side, for example, an ECG lead sticker, rather than relying on pen markings that are frequently covered by sterile drapes, may reduce the incidence of wrong site surgery.^6^ Coloured markers on the distal portion of ureteric stents could be introduced to indicate ‘left’ and ‘right’ to help prevent removal stents from the wrong side.

Human factors and ergonomics (HFE) is a scientific discipline that seeks to understand interactions between human behaviour and system safety.^10^ It is estimated that 80% of errors in the NHS are attributable to human factors at an individual or organisational level.^11^ In 2018, the Care Quality Commission (CQC) reported on NHS safety culture after visiting 18 trusts between April and June 2018 and holding focus groups with patients, staff and experts to investigate the potential contributors to NEs in the NHS. It was estimated that 96% of NEs reported in the year prior to the report should have been preventable with regular actions by humans.^12^ Global data have identified sources of general error in operating theatres to include human fallibility, miscommunication, lack of team activity, human–technology interaction and poor management of the environment.^2^, ^3^ This can be further exacerbated by poor concentration due to increased workload and long working hours.^2^ These errors can lead to non‐adherence to the protective mechanisms in place to prevent NEs, such as site marking or adherence to the WHO safety checklist.^13^ Further, in the NHS, it is common with pooled waiting lists that a surgeon examining a patient in the outpatient clinic will be different to the surgeon performing the operation at a later date. Though data are not available on the impact of this on NE frequency, it is possible that the time between booking and surgery, and the change in medical staffing, may have an impact.^14^

A recent literature review of NEs in surgery worldwide concluded that an efficient system allowing error reporting, learning from incidents and sharing of information would likely improve patient safety.^2^ Simulation sessions may also allow testing of teamwork under different pressures allowing identification of possible points of error.

There are a number of contributing factors to risk of NE occurrence that is not available in the NE database or in the literature and may warrant further investigation. It is well established that emergency surgery is associated with an increased rate of morbidity and mortality compared with elective surgery; however, the comparative rate of NEs in these two settings is not yet known or available.^15^ There may also be a link between rate of NE and time of surgery (within normal working hours vs outside of these hours), and these fields could be included in the NE reporting tool to improve data granularity. There may be a link between grade of surgeon, level of supervision (whether a trainer was scrubbed, unscrubbed but present in the theatre suite or not present) and risk of NEs. There are a number of collaborative studies including the grade of primary surgeon that are ongoing and may identify a link with surgical outcome.^16^, ^17^ Currently, there are no data available regarding grade of surgeon and risk of an NE. When considering reporting tool design, it is important to take account of burden on those uploading data to increase compliance; however, there may be value in including additional fields in the reporting tool, such as time of surgery, nature of surgery (elective vs emergency), surgeon grade, level of supervision and reporting specialty. Acquisition of this data would enable analyses that may allow organisations to direct efforts to reduce NEs. This would also allow attribution of ‘generic’ NEs such as non‐procedural NEs (e.g. drug errors) to the reporting specialty.

NHS England does not specify the specialty reporting each NE. We have therefore not included generic NEs such as retained swabs that may have been reported by urology, as we are unable to confirm the proportion of which are from urological procedures. This is a limitation of this study as the overall NE count could be significantly underestimated. We suggest that an improvement to the NHS database would be to include the reporting specialty within the database as this will allow identification of those specialties that may benefit from new interventions in an effort to reduce NEs.

## 5 CONCLUSIONS

This study demonstrates that the prevalence of NEs in urology is low. NEs are most likely to occur during procedures relating to ureteric stenting; urologists must remain mindful of the potential for wrong site surgery in these cases.

## AUTHOR CONTRIBUTIONS

Jerocin Vishani Loyala, Andrew Ang and Billy Down collated data and wrote the first draft of the manuscript. Sarah A. Howles oversaw the project, reviewed data and modified the drafted manuscript.

## CONFLICT OF INTEREST STATEMENT

Sarah A. Howles is a Wellcome Trust Clinical Career Development Fellow. This research was funded in part by the Wellcome Trust 220 668/Z/20/Z. For the purpose of Open Access, the author has applied a CC BY public copyright licence to any Author Accepted Manuscript (AAM) version arising from this submission. Jerocin Vishani Loyala, Andrew Ang and Billy Down have no conflicts of interest to declare.

## ETHICS STATEMENT

This research was undertaken using anonymised data sets that exist within the public domain and accessed via the National Reporting and Learning System (NRLS) website and therefore does not require ethical approval.
